# The impact of paramedics working in primary care teams on other professionals and patient experiences: a qualitative study

**DOI:** 10.3399/BJGPO.2024.0152

**Published:** 2025-08-13

**Authors:** Georgette Eaton, Stephanie Tierney, Geoff Wong, Veronika Williams, Julia Williams, Kamal R Mahtani

**Affiliations:** 1 Nuffield Department of Primary Care Health Sciences, University of Oxford, Oxford, United Kingdom; 2 School of Nursing, North Bay, Canada; 3 School of Health and Social Work, University of Hertfordshire, Hatfield, United Kingdom

**Keywords:** qualitative research, paramedics, primary health care

## Abstract

**Background:**

Paramedics are among the professional groups identified in recent policy initiatives aimed at addressing the unsustainable workload and workforce crises in primary care. Their support aims to enhance patient access to care and alleviate the burden of workload pressures.

**Aim:**

To explore the impact of paramedics working in primary care on primary care teams and the experiences of patients who have a clinical consultation with a paramedic in primary care.

**Design & setting:**

A qualitative study using focused observations and interviews, involving 15 geographically dispersed sites across the UK.

**Method:**

Data were collected between May 2022 and January 2023, incorporating 60 semi-structured interviews and 60 hours of observations of paramedics. Transcripts were thematically analysed.

**Results:**

Patients, GPs, and other staff in primary care perceive that the paramedic role enhances healthcare availability in primary care by increasing workforce capacity. This is especially prevalent when paramedics work in a clinical capacity that complements the GP role. However, successful integration into the primary care team relies on paramedics having sufficient clinical experience and receiving clinical supervision from GPs. Patients are trusting of the paramedic role when they have positive clinical consultations.

**Conclusion:**

Paramedics have potential to improve access to the primary care workforce. However, attention to supportive transition processes (such as clinical supervision) are required for the paramedic to successfully be integrated into the primary care team.

## How this fits in

Paramedics have been outlined in workforce policy as key contributors to alleviating workforce pressures within primary care. However, the impact of paramedics’ integration into primary care teams and the experiences of patients who engage in clinical consultations with them has not previously been explored. The findings of this study suggest that the paramedic role can enhance healthcare availability in primary care by increasing workforce capacity when attention is given to their transition into the primary care team. It is important for primary care employers and GPs to be aware of factors relating to transition in order to achieve successful integration of paramedics into the primary care team.

## Introduction

In response to the unsustainable workload and workforce crises in primary care, policy has been introduced across the NHS outlining the use of additional roles to improve access to primary care.^1–4^ Research has focused on the implementation of some of these roles through the Additional Roles Reimbursement Scheme (ARRS) in England,^5,6^ finding that the scheme has expanded the range of expertise in primary care without significantly reducing the burden on GPs. Paramedics are one of the professional groups included in ARRS in England. However, paramedics have been known to be working in primary care across the UK since 2002,^7^ long before the implementation of this policy. Initial research indicated that the employment of paramedics in primary care roles lacked standardisation across the UK^8^ and subsequent studies have further detailed the specific clinical roles that paramedics undertake in primary care settings^9^ and their cost-efficiency.^10^ However, concern exists regarding the integration of paramedics into primary care, specifically regarding potential issues around role duplication and role substitution of other well established clinical roles within the workforce.^11^ There is limited research regarding the experiences of primary care teams within which a paramedic is employed, and the experiences of patients.

This study aimed to explore the perceived impact of paramedics working in primary care on primary care teams and the experiences of patients who have a clinical consultation with a paramedic in primary care.

## Method

### Design

The study design consisted of focused observations^12^ and semi-structured interviews with paramedics working in primary care in the UK, patients who had received a consultation with the paramedic, and healthcare professionals and administrative staff working with the paramedic.

### Sampling

Each paramedic was regarded as a ’case’, serving as the central focus for data collection. Additional data were gathered by conducting interviews with three key individuals associated with the ’case’: one GP, one other healthcare professional or administrative team member, and one patient or carer. The sampling framework was purposive, using a maximum variation approach^13^ in order to sample individuals who differed in terms of time as a paramedic, length of time in primary care, employment type in primary care, job title and level of education, and consider the extent to which these factors impacted paramedic integration into primary care teams.

Previous work investigating roles of healthcare professionals in primary care in England^14^ has outlined that 12 cases can provide rich enough data to explore role implementation. However, as this study was UK-wide, a sampling framework of 15 cases (paramedics) was considered more suitable to demonstrate the breadth of practice across the UK.^15^ Table 1 outlines the eligibility criteria for participants.

**Table 1.** Eligibility criteria for participants

Inclusion criteria	Exclusion criteria
Participants can converse in English	Participants who cannot converse in English
Participants are willing and able to give informed consent	Participants aged <18 years
They will fit one of the following three profiles: Paramedics working in primary care within the UK Adult patients in the UK who have had contact with a paramedic within a 3-month window before the start of data collection around the case (paramedic) Healthcare professionals and administrative staff employed in primary care in the UK or who work alongside a paramedic	Adult patients experiencing significant psychosocial difficulties that would make it unreasonable to invite them to take part in the research

### Recruitment

Paramedics working in primary care were purposively sampled from participants responding to a previous cross-sectional survey who agreed to be approached for involvement in future research.^9^ In addition, adverts were sent to primary care teams by Clinical Research Networks (CRNs) in England, health boards within Wales, Northern Ireland’s Clinical Research Network (NICRN), and regional health boards within Scotland. These groups coordinate and support the delivery of research within their geographical locality, and act as a gateway to research for health services within that locality. Paramedics who took part were considered as cases, who would enable connection to other data sources (for example, patients and healthcare professionals). Individuals were contacted by email to confirm their willingness to participate, their eligibility regarding the sampling criteria (Table 1), and to arrange an initial conversation with them and their primary care employer to determine their willingness to participate.

Once a site was selected, the primary care provider sent a letter (including a participant information sheet) to registered patients. This letter provided details about the timing of the focused observations and invited patients to take part in the interviews following consultation with the paramedic within a 3-month period.

Recruitment of other healthcare professionals and administrative staff for semi-structured interviews, such as GPs, nurses, pharmacists, and administrative staff, followed a snowballing approach initiated through contacts with the participating paramedics (cases). Participants interested in being interviewed reached out to GE for an initial conversation. Following this, a mutually convenient date was arranged for the interview, conducted either face to face, via Microsoft Teams or via telephone, depending on the participant’s preference.

All participants provided informed consent, either written or verbally, before observation and/or interview.

### Data collection

Paramedics were observed for 4 hours undertaking their primary care role. This role included practice-based consultations, telephone consultations, and home visits. Observations focused on gathering data relating to three abstract categories developed from previous work: expectations of paramedics working in primary care; integration of paramedics into primary care teams; and their role and responsibilities within these teams.^11^ An interview schedule was developed based on existing literature and the study aims. This included a list of questions suitable for each participant group (Supplementary Text 1). Questioning for patients was developed by the patient participation group affiliated with this research, and interviews were piloted with member representatives of the associated stakeholder group. GE undertook observations and collected the interview data between May 2022 and January 2023.

### Analysis

Interviews were audio-recorded, de-identified, and transcribed. Transcriptions and field notes from the focused observations were analysed using semantic level, inductive reflexive thematic analysis^16^ in NVivo (version 12), and the results integrated. Analysis focused on understanding and explaining identified patterns in the data^17^ by drawing on existing conceptual frameworks identified in previous research.^11^ The analysis was conducted by the authorship team, and feedback was sought from the stakeholder group, which included patients and members of the public affiliated with this research.

During analysis, we adopted a reflexive approach by continuously reflecting on our positionality, potential biases, and the ways the presence of the researcher (GE) may have influenced both the observations and interviews. Regular team discussions and reflexive journaling were employed to ensure that our interpretations remained grounded in the participants’ experiences while acknowledging our role in the research process.

## Results

Fifteen focused observations were undertaken of paramedics working in nine Clinical Research Networks (CRNs) in England, two health boards in Scotland, two health boards in Wales, and one health board in Northern Ireland. This included a range of settings, encompassing rural, urban, and coastal locations. Of the 15 paramedics, eight were employed directly by the practice, and of these two worked part-time alongside other roles in education. Four paramedics were employed full-time by the primary care network; and three worked on a rotational arrangement between the local ambulance service and the primary care provider. Further information is outlined in Table 2. Only three practices in England accessed ARRS funding to facilitate paramedic employment. Sixty interviews with participants (as shown in Table 3) were conducted. Observations lasted a maximum of one session (4 hours) and interviews lasted on average 23 minutes (ranging from 5–58 minutes). Triangulating interview and observational data allowed us to check for consistency across sources, thereby strengthening the credibility of the research.

**Table 2.** Details of paramedic cases

Site	Participant type	Length of time as a paramedic (years)	Length of time in primary care (years)	Employment type	Geographical information
UK 1	Paramedic practitioner	14	6	PCN	Urban: town
UK 2	Paramedic practitioner	10	4.5	Direct employment	Rural: villages
UK 3	Specialist paramedic	27	5	Direct employment	Urban: city
UK 4	Paramedic	20	9 months	PCN	Coastal: town
UK 5	Paramedic	2.5	4 months	PCN	Urban: city
UK 6	Paramedic practitioner	8	11 months	PCN	Urban: city
UK 7	Home visiting paramedic	12	3	Direct employment	Coastal: town
UK 8	Urgent care practitioner (paramedic)	19	3	Rotational (ambulance trust and primary care)	Rural: villages
UK 9	Paramedic practitioner	14	4	Direct employment	Rural: town
UK 10	Paramedic practitioner	9	3	Direct employment	Rural: town
UK 11	Advanced paramedic practitioner	18	6	Rotational (ambulance trust and primary care)	Rural: villages
UK 12	Advanced paramedic	30	22	Direct employment	Coastal: rural
UK 13	Advanced paramedic practitioner	11	4	Direct employment	Urban: city
UK 14	Advanced paramedic practitioner	19	1.5	Rotational (ambulance trust and primary care)	Coastal: town
UK 15	Paramedic practitioner	23	5	Direct employment	Rural: town

PCN = primary care network

**Table 3.** Participant demographics from focused observations and interviews

Participant type		Number
Paramedic	Advanced paramedic	1
	Advanced paramedic practitioner	3
	Home visiting paramedic	1
	Paramedic	2
	Paramedic practitioner	6
	Specialist paramedic	1
	Urgent care practitioner (paramedic)	1
Patients	Patient	13
	Patient carer	2
GPs	GP partner and trainer	1
	GP partner	3
	GP trainer	2
	Salaried GP	9
Other healthcare professionals and administrative staff	Administrative support staff	1
	Advanced nurse practitioner	3
	Clinical pharmacist	1
	Healthcare assistant	1
	Home visiting district nurse	1
	Practice manager	4
	Practice nurse	1
	Receptionist	3

**Figure 1.**
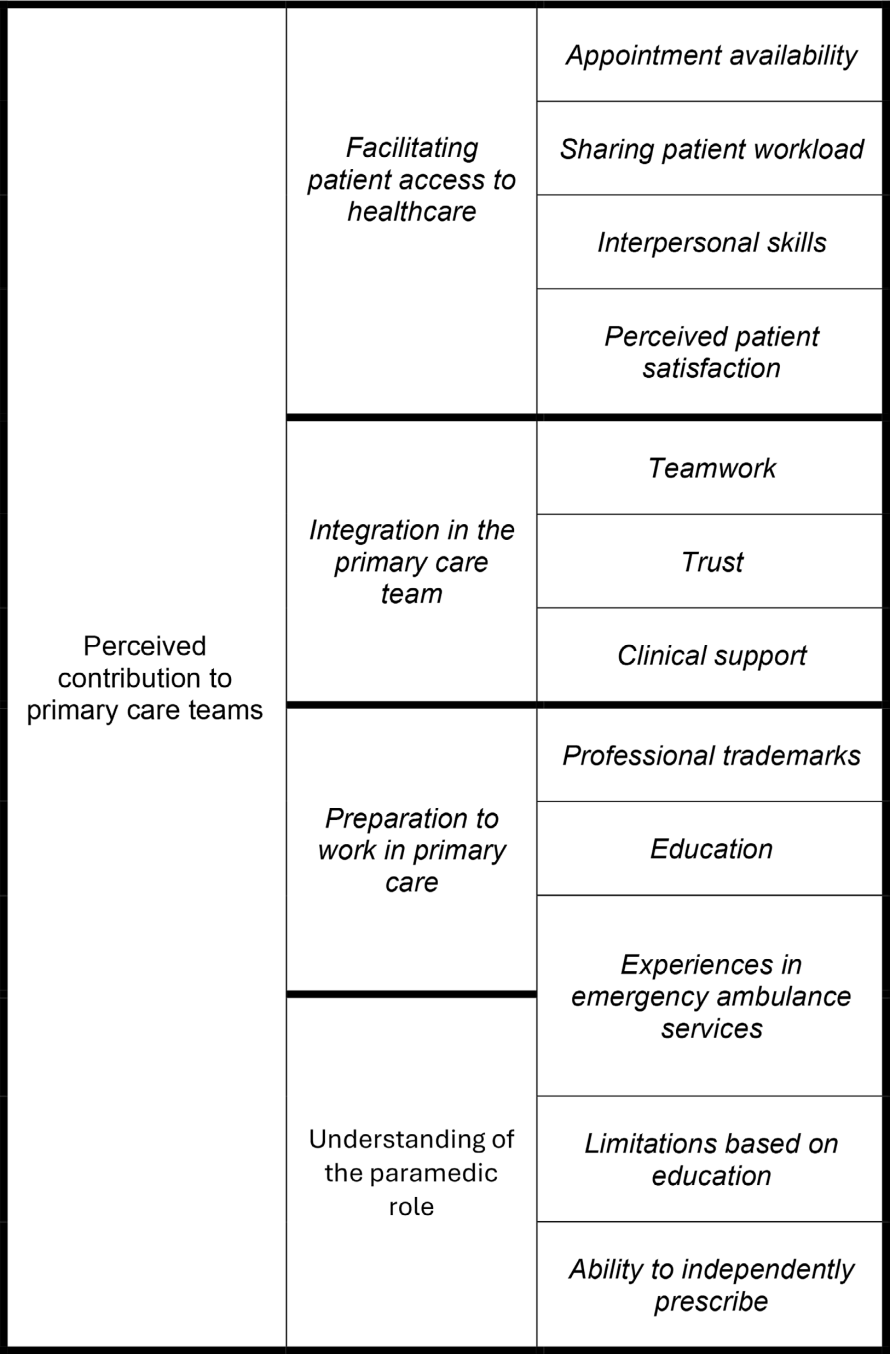
Conceptual framework of findings

The findings are evaluated within our previously established conceptual framework that highlights the importance of understanding different expectations regarding how paramedics can contribute and operate in primary care.^11^ We treated this framework as a context from which to consider information regarding how the paramedic role can be integrated into primary care through their perceived contributions to primary care teams, as illustrated in Figure 1.

### Facilitating patient access to health care

Of the 15 patients interviewed, 10 were not informed in advance that they were seeing a paramedic for their consultation in primary care, but all reported being made aware at the start of their consultation that they were seeing a paramedic.

GPs generally felt that paramedics were *‘very valuable to general practice’* (UK10-02: GP trainer), where several practices found *‘it’s got to that point where we notice when* [the paramedic is] *off, you know and I think that’s the greatest compliment that you can get’* (UK2-03: GP partner). Paramedics felt they were able to contribute to the primary care team by reducing workload on other professionals in the practice. This sentiment was echoed by GPs who felt the role *‘had a positive impact’* in providing *‘on the day access’* (UK5-02: Salaried GP):

*‘… when he takes home visits off us — it’s physical time. When he takes patients, we probably still end up seeing as many patients ourselves, but it improves patient access and patient satisfaction, which has a massive impact on morale day-to-day.’* (UK8-02: Salaried GP)

There was a sense from patients that paramedics working in primary care increased the availability of appointments for patients to be seen, resulting in improved access overall:

*‘I know that GPs are few and far between to the number of patients that need them. So it was great to have her as an asset really*.’ (UK6-02: Patient)

This sentiment was also echoed by one paramedic who *‘describes himself as a mop — mopping up what comes in late that cannot get on a list’* (UK7: Fieldnotes).

Patients who were interviewed consistently emphasised the importance of the interpersonal skills of the paramedic. It became apparent during focused observations that the paramedics were quick to build rapport with patients they saw, typically adopting an informal approach with the patient:

*‘I get the sense that the paramedic is very integrated both within the practice and the local community. One thing I noted during every patient encounter was how patients remarked on his interpersonal skills — he was friendly, a listening ear, professional, kind, understanding. He is accepted in his role, and the community.’* (UK2: Fieldnotes)

Important interpersonal skills reported by patients included listening, empathy, and adopting a holistic approach to the individual:

’… *he’s a great mannerism, he’s got great interaction, especially for mum who’s older and loves a bit of craic ... He’s always smiling and that means a lot.’* (UK2-04: Patient carer)

Even when paramedics needed to consult a senior clinical colleague or GP during an appointment, patients did not perceive this as duplication. Instead, they reacted positively to the paramedic seeking additional guidance. This collaborative approach to decision making fostered a sense of confidence and trust in the paramedic among patients:

*‘… you know she’ll go and ask if she needs help, you know, without a doubt.’* (UK1-04: Patient)

When considering the satisfaction of patients they saw, paramedics outlined that *‘feedback has been positive’* (UK4-01: Paramedic) and regarding their role in primary care, where the increase in healthcare access afforded them to offer slightly longer consultations than their GP counterparts:

*‘…* [patients] *have said thank you for treating me as a person and not just as a patient … it’s having the time looking at that whole picture and treating them holistically.’* (UK6-01: Paramedic practitioner)

However, there were concerns about the ethos underpinning the nationwide implementation of the role:

*‘The government are using it as a sticking plaster to replace GPs and I don’t think that works. They should be there to complement the role we do … But I don’t think they’re a replacement. I think patients would probably agree with me as well.’* (UK5-02: Salaried GP)

### Integration in the primary care team

Practice managers and receptionists noted that paramedics were well integrated into their primary care teams, offering valuable support beyond clinical tasks: *‘he’s forever helping out the reception teams or some of their queries …’* (UK3-03: Practice manager). This was also observed during focused observations:

*‘There is a joviality to the conversation and, whilst the paramedic takes the brunt of some of the jokes, he contributes to the banter. This is clearly a team that knows each other and works well together*.’ (UK 9: Fieldnotes)

A similar sentiment was also expressed among the clinical team members, who outlined that the paramedic was *‘part of our normal team’* (UK15-04: Practice nurse), with an appreciation for the contribution of the profession to primary care:

*‘... a really vital part of our team — we really enjoy working with her — partly because we looked at things a little bit differently.’* (UK6-04: Advanced nurse practitioner)

For GPs, effective collaboration with paramedics was founded on a relationship built on trust. Despite paramedics being a registered profession, many GPs felt that *‘the overall responsibility lands with the doctor, not with the paramedic’* (UK3-04: GP partner). Even with paramedic registration, the belief persisted that *‘… a doctor’s responsibility’s greater’* (UK9-03: Salaried GP). As a result, trust between GPs and paramedics was essential for the latter to contribute to the healthcare team. One GP emphasised:

*‘… in reality if* [the paramedic] *sent me ten prescriptions in a day, I can’t see those ten patients myself. It completely invalidates the point of having her. So, we know that on the ground you’ve kind of got to put an element of trust into the person you’re working with.’* (UK5-03: Salaried GP)

Building trust in the paramedic’s abilities was a gradual process that required consistent clinical support and supervision. Ten out of the 15 paramedics interviewed benefitted from regular opportunities for direct clinical supervision, with the remaining five having access to indirect supervision in terms of access to support when needed. Paramedics linked the provision of clinical supervision to the development of a trusting relationship with the GPs they worked with:

*‘… when we first started, and I was having to debrief — that’s how they built their confidence in me …*’ (UK9-01: Paramedic practitioner)

However, dedicating time during GPs’ practice hours to provide this oversight increased the GP workload burden:

*‘... if you put in enough work with them to start off with, you’ll get a lot out of them, it’s just having the patience to do it.’* (UK13-02: GP trainer)

GPs who reported not having this trusting relationship noted that the paramedics would *‘bring some work to you as well’* (UK3-04: GP partner), which was the antithesis of what such additional roles were posited to contribute.

While the paramedics observed were well integrated with primary care teams, working in primary care was considered to *‘be a lonely existence’* (UK15-04: Paramedic practitioner), owing to infrequent interactions with other paramedics in the same clinical setting.

### Preparation for paramedics to work in primary care

The preparation for paramedics to work in primary care encompasses a range of training, education, and experiential learning activities that may be used to address the specific requirements and challenges of working in this environment, and how these experiences were perceived by the teams they joined.

Paramedics believed that their previous experiences in emergency ambulance services not only prepared them for primary care but also represented the distinctive qualities of their profession. They saw skills such as *‘critical thinking and balancing risk, risk management’* (UK10-01: Paramedic practitioner) as attributes that made them proficient in primary care. Additionally, their ability to assess the social environment and conduct social assessments, gained through *‘experience of going into people’s houses and picking up on the social environment’* (UK14-01: Advanced paramedic practitioner) were regarded as hallmark features of their profession. Across all GPs interviewed, there was a consensus that work in the ambulance service prepares paramedics to work in primary care because they were used to *‘fast-paced environments … working under pressure … dealing with urgent cases’* (UK10-02: GP partner).

Length of time as a paramedic was considered to be advantageous to paramedics entering primary care, while lack of experience was considered as an important barrier for paramedics to work in primary care:

*‘I would want a paramedic plus ACP training on the top or a paramedic who’s been working for a good 5/10 years you know on the ambulances so has had lots of experience.’* (UK3-04: GP partner)

*‘... don’t think somebody whose newly qualified as a paramedic would … have the skills that* [our paramedic has] *got by far. They need that ground out on the road and we need paramedics out on the road. We don’t need them all in primary care.’* (UK15-04: Practice nurse)

This was echoed by paramedics:

*‘I think there is definitely something about being a good core paramedic before transitioning … you’ve got to know what your profession is and where you’ve come from.’* (UK11-01: Advanced paramedic practitioner)

Across all sites, paramedics noted the need for additional education to work in primary care. However, paramedics noted that structured education on its own was not enough, and any additional education needed to be supported by experiential learning. While there was a transference of some skills from the ambulance service to primary care, primary care was considered *‘a massive learning curve’* (UK15-01: Paramedic practitioner). Paramedics were said to *‘have to adapt and make it work’* (UK9-01: Paramedic practitioner), which itself was considered to be another paramedic skill.

### Understanding of the paramedic role

There was a perception among patients that paramedics were *‘very, very knowledgeable’* (UK104: Patient), *‘very highly trained’* (UK2-04: Patient), and that *‘paramedics do everything’* (UK13-03: Patient) because of their experience with emergency situations from the ambulance service. However, on being told they were seeing a paramedic in primary care, one patient outlined their *‘very first reaction was slight panic that there should be something to worry about’* (UK6-02: Patient).

Non-clinical staff in primary care displayed limited understanding of the paramedic profession, with their knowledge largely shaped *‘from seeing like Casualty or something’* (UK1-03: Receptionist). However, working alongside the paramedic, staff exhibited growth in their comprehension of the role.

For GPs, patients, and both clinical and non-clinical staff in primary care, limitations in the role were outlined, particularly around patient groups they would not typically see through the course of their work in emergency ambulance services, and therefore saw less in primary care. This included women’s and men’s health, palliative care, assessment of infants, and specific long-term conditions. One patient outlined:

*‘… I feel that they wouldn’t be so sure is say as a female and you’re wanting to get your breasts checked or you had gynaecological problems or something. I think that then is out of their league. Personally, to me I would say that I’d need a GP for that side of things.’* (UK8-04: Patient)

GPs also perceived paramedics as primarily trained for emergency care, which was seen as limiting the breadth of their knowledge needed for work in primary care:

’… *they are more than welcome to join in and give a hand, but it is not really what they have been trained to do …’* (UK11-04: Salaried GP)

The focus on emergency medical care was also perceived to limit the paramedics’ critical thinking and decision-making abilities, particularly with complex patient cases:

*‘They obviously haven’t had the training of a GP … they haven’t got that thinking process of sometimes bringing it all together, so they can often deal with one condition, say, but maybe can’t bring all of it together without support …’* (UK10-02: GP trainer)

The single biggest limitation reported by GPs, clinical and non-clinical staff surrounded paramedics who were not yet working as independent prescribers:

*‘I think it would be beneficial for her as well if she could do the prescribing rather than having to keep asking people.’* (UK6-04: Advanced nurse practitioner)

Even when paramedics could prescribe, limitations in their prescribing scope resulted in some frustration when it impacted other members of the primary care team.

## Discussion

### Summary

This study examined the impact of paramedics working in primary care on both primary care teams and the experiences of patients who had clinical consultations with paramedics. The findings indicate that paramedics in primary care can enhance healthcare availability by increasing workforce capacity. However, successful integration within the practice is essential for this to occur. Both paramedics and GPs emphasised the importance of clinical support and supervision. Paramedics were seen as complementing the GP role, without duplicating or infringing on their roles. This stands in contrast to recent studies that highlighted unintended role expansion among clinicians employed under the ARRS.^6^ Patients who consulted with paramedics reported positive experiences, particularly praising the paramedics’ strong interpersonal skills. This high level of patient satisfaction was largely attributed to the paramedics’ effective use of these skills during consultations, rather than the consultation outcome.

### Strengths and limitations

While other studies have examined the overall influence of ARRS roles on the primary care workforce in England,^18^ this research focuses on one professional group within this funding scheme employed across the entire UK, including devolved nations where ARRS does not apply. The strength of this work lies in its potential transferability to different regions, as the diverse sample reinforces the applicability of the results.

This research has some limitations. First, its case-based approach captures a snapshot of paramedic practices across 15 UK sites, which may not represent the entire paramedic workforce in all home nations. However, the consistency in working practices across these sites is encouraging. Second, despite efforts to ensure variation in the sample, the nature of the research may have attracted participation primarily from primary care settings with positive working relationships with their paramedics. This could explain the overall positive findings in the results. In addition, all patient interviews expressed favourable views of the paramedics’ role in primary care, and there are possibly instances of poor experiences that were not captured. Additionally, patient interviews were the briefest among all conducted, likely because they focused on the specific event of a consultation rather than broader life experiences. Interviews with other primary care staff were selected from those who had the most interactions with paramedics, potentially including staff members with positive working relationships with paramedics, making them more inclined to participate in the study.

### Comparison with existing literature

The importance of clinical supervision and integration within the primary care team has been well documented for additional roles within primary care.^5,6,9,11,18–20^ This study highlights that paramedics manage the ambiguity they face when transitioning from the ambulance service to primary care by seeking clinical support, particularly from GPs. Interviews with GPs and other clinical staff outlined that there were no concerns regarding role substitution or conflict when paramedics were employed, which was a key finding from a realist review,^11^ but do outline that the paramedic role, while improving patient access, can increase their own workload.

Patients who had clinical consultations with paramedics in primary care consistently expressed positive opinions, echoing previous findings,^21^ corresponding to paramedics’ own perceptions of patient satisfaction.^9^ This contrasts sharply with the annual GP Patient Survey for England, which reported a significant decline in patient satisfaction, especially regarding appointment accessibility.^22^ The ease of access to primary care provided by paramedics likely contributes to the high levels of satisfaction observed in this study.

### Implications for research and practice

In order for paramedics to successfully integrate into primary care teams, they need support to transition into this setting. Employment of paramedics with sufficient clinical experience in ambulance service work enables clinical range. This experience, coupled with a higher level of education, is important if they are to effectively complement the GP role. This research highlights that the role of paramedics in primary care is highly variable. When GPs or other primary care staff do not fully understand the scope of role a paramedic can offer, they fail to recognise the valuable contributions paramedics can make within their team. This lack of understanding impedes the integration of paramedics into the primary care team and limits their effective utilisation in the workforce.

For GPs to build trusting relationships with paramedics they clinically supervise initially requires a significant time investment, and our data suggests time needed to support decreases once trust is established, allowing the paramedic to work more autonomously. Patients trust paramedics because they are part of the primary care team, and this trust is further solidified when paramedics escalate care to the GP. When patient expectations are met, through longer appointment times or the establishment of therapeutic rapport with the paramedic, they feel reassured about the quality of care received. This reassurance leads to higher satisfaction with the appointment and a willingness to consider future appointments with the paramedic.

Further research is needed to evaluate the cost-effectiveness of paramedics in primary care and to determine their impact on GP productivity. Investigating how the paramedic role influences heavy GP workloads would be particularly valuable, as the supervision needed as these roles transition into the primary care workforce are often overlooked in policymaking.

## Data

The dataset relied on in this article is available from the corresponding author on reasonable request
